# Confocal laser microendoscopy characterization of vascular features in facial basal cell carcinoma: a comparative analysis of tumor and adjacent healthy tissue

**DOI:** 10.1007/s10103-026-05016-x

**Published:** 2026-09-09

**Authors:** Flurin Müller-Diesing, Matti Sievert, Marc Aubreville, Stephan Hackenberg, Bharat Panuganti, Maurice Klein, Nils Porsche, Agmal Scherzad, Manuel Stöth, Philipp Winnand, Markus Wirth, Miguel Goncalves

**Affiliations:** 1 Department of Otorhinolaryngology, Universitätsklinikum Aachen, Aachen, Germany; 2 Department of Otorhinolaryngology, Head and Neck Surgery, Universitätsklinikum Erlangen, Erlangen, Germany; 3 Flensburg University of Applied Sciences, Flensburg, Germany; 4 Department of Oto-Rhino-Laryngology, Head and Neck Surgery, Universitätsklinikum Würzburg, Würzburg, Germany; 5 University of Alabama at Birmingham Hospital, Birmingham, USA; 6 Department of Oral and Maxillofacial Surgery, Universitätsklinikum Aachen, Aachen, Germany

**Keywords:** Basal cell carcinoma, CLE, Tumor vessels, Optical biopsy

## Abstract

An initial application of Confocal laser microendoscopy (CLE) in basal cell carcinoma (BCC) was promising, yet data in this field are still limited. Tumor vasculature, which can be clearly visualized by CLE, differs significantly from that of normal tissue, but has not yet been systematically analyzed as a target of CLE in BCC. By systematically assessing vascular features, we aim to define diagnostic criteria that support a more standardized and objective use of CLE in BCC. CLE imaging was performed in nine patients with histologically confirmed facial BCC. Forty artifact-free vascular sequences (20 BCC, 20 healthy skin) were analyzed using five predefined criteria independently rated by three blinded reviewers. Diagnostic accuracy, sensitivity, specificity, and interrater agreement were calculated. Normal skin showed regular, well-defined, round vessels of uniform size, whereas BCC demonstrated heterogeneous, dilated, and elongated vessels with indistinct borders and variable flow. Mean sensitivity and specificity were 91.7 ± 4.7% and 86.7 ± 6.2%, respectively, with substantial interrater agreement (κ = 0.77). Vascular features in CLE sequences supported the differentiation between BCC and healthy skin. Incorporating vascular criteria into CLE-based scoring systems appears promising for a more standardized differentiation of BCC from healthy skin. Further studies with larger patient cohorts are warranted to validate these preliminary findings and assess their clinical applicability. Not applicable, as this study is a retrospective analysis.

## Introduction

The management of basal cell carcinomas (BCC) in the facial region represents a distinct clinical challenge. Although BCCs typically exhibit slow growth and rarely metastasize, complete surgical excision is essential, as incomplete resection substantially increases the risk of recurrence [1]. Without appropriate treatment, BCC may progress to extensive ulcerations with destruction of surrounding structures, creating not only aesthetic disfigurement but also considerable difficulties in clinical care. In this context, surgical excision is generally considered the treatment of choice [2]. However, reported rates of complete (R0) resection are highly heterogeneous, and incomplete excisions have been described with an average frequency of approximately 11% [3]. Moreover, the face is particularly vulnerable both functionally and cosmetically: even minor tissue loss, especially on the nose or ear, can considerably limit reconstructive options and outcomes. These considerations highlight the potential value of noninvasive diagnostic methods such as optical biopsy techniques.

Confocal laser endomicroscopy (CLE), a well-established imaging technique, is increasingly being applied to further tumor entities and clinical specialties, expanding its role in oncologic diagnostics. In a pilot feasibility study, our group demonstrated that CLE is fundamentally capable of differentiating BCC from normal skin [4]. CLE relies on detection of fluorescein fluorescence following intravenous administration, excited and captured by the laser probe, producing real-time in vivo images of superficial tissue layers (penetration depth 55–65 μm) [5]. A first analysis suggested that features such as tissue homogeneity and cell size may serve as markers to distinguish between BCC and healthy tissue [4]. To establish CLE in routine practice, however, further characterization of specific morphological criteria and reduction of inter-observer variability are required.

One aspect not yet systematically studied in BCC is vascular morphology. Angiogenesis in tumors differs markedly from that in normal tissue. Uncontrolled and disorganized tumor growth generates poorly vascularized, hypoxic regions. Hypoxia, together with direct secretion of pro-angiogenic mediators by tumor cells, drives excessive local angiogenic signaling. This results in aberrant vessel formation, endothelial maturation defects, and further perpetuation of hypoxia. Consequently, tumor vasculature is characterized by highly permeable, irregular, and structurally abnormal vessels [6].

Because CLE visualizes tissue based on intravenous administration of fluorescein, it provides an opportunity to study vascular features directly [5]. Prior studies in other tumor types, including head and neck squamous cell carcinoma (HNSCC), have suggested that vascular patterns may offer additional diagnostic value [7–10]. Demonstrating distinct vascular characteristics in BCC compared with healthy skin could therefore establish vascular morphology as a novel diagnostic criterion for the use of CLE on BCC.

The aim of the present study is to further systematize CLE in dermatologic oncology by investigating vascular features in BCC. Specifically, we seek to define initial morphological criteria that distinguish BCC from normal tissue and thereby enhance diagnostic reliability independent of observer expertise. To our knowledge, this is the first study to directly compare CLE-visualized vascular patterns in BCC and healthy skin.

## Materials and methods

### Study design

In this study, previously acquired CLE-based vascular imaging data with histologically confirmed correlations (BCC or healthy skin) were systematically reviewed and analyzed by the first author. Subsequently, the data were independently assessed in a blinded fashion by three investigators.

## CLE examinations

CLE examinations were performed immediately prior to surgical excision. Patients with preoperatively histologically confirmed facial BCC received 2.5 mL of intravenous fluorescein. If image quality declined during the examination, an additional 2.5 mL fluorescein was administered to allow continuation of the procedure. Using a handheld CLE probe, guided like a pen, the operating surgeon sequentially examined the tumor and adjacent clinically uninvolved perilesional skin within the same anatomical region. Distinct video sequences were recorded from different subregions.

Immediately thereafter, tumor resection was performed, and corresponding tissue areas were submitted separately for histopathological analysis, enabling correlation of CLE recordings with histologically verified tissue (BCC and adjacent normal-appearing skin from the same surgical specimen).

The reference “healthy skin” therefore consisted of macroscopically uninvolved perilesional tissue obtained in close proximity to the tumor site.

## Video processing

Video sequences were processed using Cellvizio Viewer software (version 1.6.2.A; Mauna Kea Technologies, Paris, France). To reduce selection bias, all available CLE recordings obtained from histologically verified tumor and healthy tissue sites were screened systematically. From the complete available recordings, all video sequences displaying vascular cross-sections or vascular structures were extracted for analysis, while video material substantially compromised by motion artifacts or poor image quality was not included. Duplicate video sequences of the same tissue region were also excluded.No exclusion was based on vascular morphology. The final dataset consisted exclusively of artifact-free vascular sequences suitable for blinded assessment.

## Characterization of vascular features

An unblinded review of the sequences was performed by the first author. Based on these observations and prior clinical experience with CLE in BCC and other tumors, characteristic vascular features for BCC and normal tissue were defined, and five criteria were established to allow differentiation between vessels from healthy tissue and BCC (Table 1). A briefing package, consisting of four representatives still images and brief explanatory notes, was provided to the expert reviewers.

**Table 1:** Vascular features. Listing of the five criteria assessed in a blinded fashion by three reviewers

Number	Criterion
1	Vessels with oval or round morphology
2	Multiple Vessels of similar morphology
3	Narrow, elongated vessels
4	Extensive vessels without clear defined margins
5	Visible, accelerated flow

## Blinded CLE evaluation

The selected video sequences were independently and blindly evaluated by three investigators, each with a minimum of five years of experience in CLE examinations [4, 7–9, 11]. For each sequence, the reviewers first assessed the presence or absence of the five predefined vascular characteristics. Subsequently, they classified the vessels as either BCC or healthy. All evaluations were performed exclusively based on the provided briefing package, with the reviewers blinded to the histopathological results.

### Statistical analysis

Statistical analyses were conducted using SPSS Statistics version 26.0 (IBM, Armonk, NY, USA). For each reviewer, accuracy, sensitivity, specificity, positive predictive value (PPV), and negative predictive value (NPV) were calculated individually, and mean values across all three reviewers were then determined. Inter-rater reliability was assessed using Fleiss’ kappa.

To examine the five predefined vascular criteria, Cochran’s Q and McNemar’s tests were applied to compare the occurrence of the criteria with one another, separately in tumor and healthy tissue.

## Results

### Epidemiological and technical data

In total, CLE sequences from nine patients were included. Among these, two patients had a BCC located on the nose and seven patients had a BCC on the auricle. From the raw data comprising 9888 images, with corresponding histopathological correlation, 40 artifact-free vascular sequences comprising a total of 2904 images were obtained. Half of the sequences represented BCC, the other half healthy tissue.

### Characterization of vascular features

**Fig. 1:**
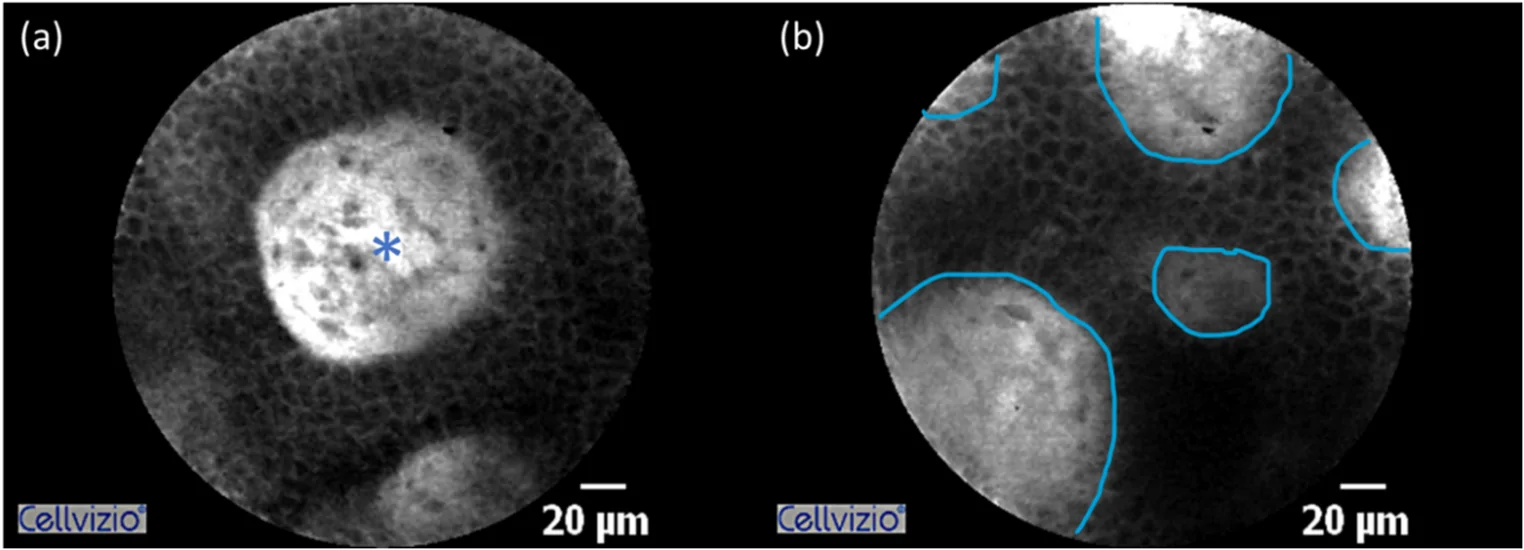
Vascular structures in healthy tissue. (**a**) Well-demarcated, rounded vessel cross-section (blue asterisk). (**b**) Several similarly shaped, rounded, well-defined vessel cross-sections, closely adjacent (blue markings)

Based on the CLE vascular sequences, the first author identified characteristic vascular features in healthy tissue and in BCC. In healthy tissue, vessel cross-sections were oval or round with sharply defined borders. Multiple similarly shaped vessel cross-sections were frequently observed in close proximity (Fig. 1).

**Fig. 2:**
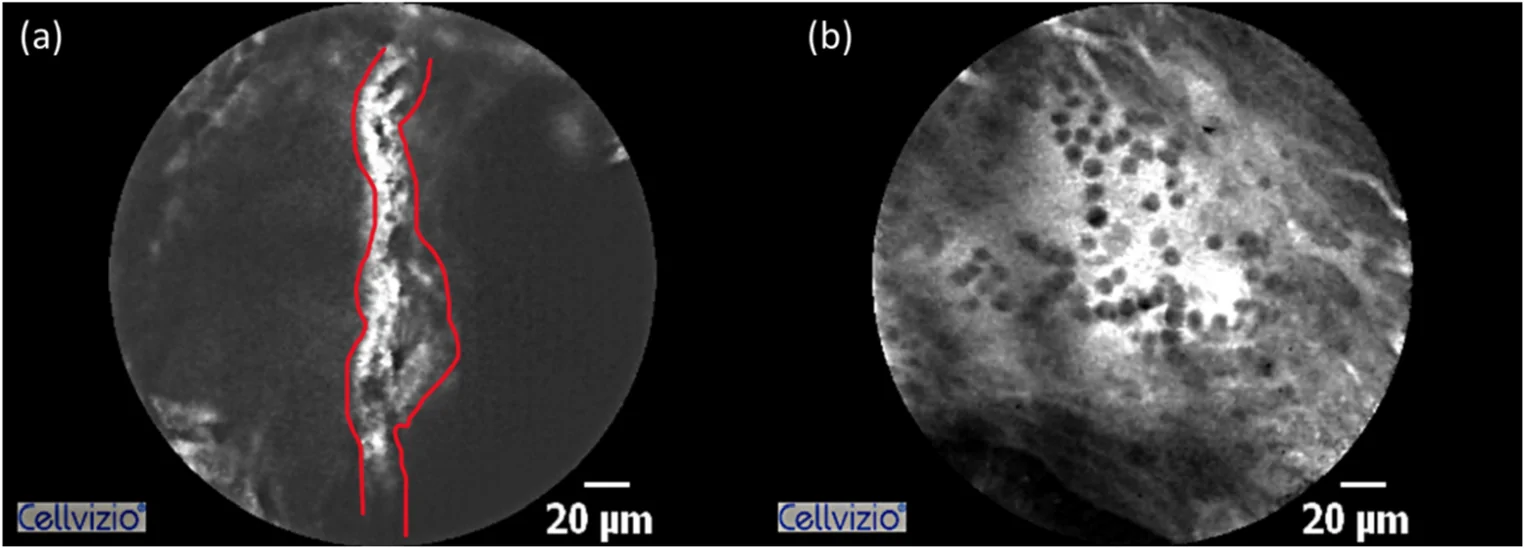
Vascular structures in BCC. (**a**) Narrow, elongated vessel (red markings). (**b**) Dilated vessel with poorly demarcated borders, showing variable flow velocities in the corresponding video sequence

In BCC, vessels appeared more heterogeneous. They were either elongated and narrow or markedly dilated with poorly defined borders. Vessel diameter, shape, and size varied considerably, and vessel density was more irregularly distributed throughout the tissue. In some vessels, accelerated flow of individual blood cells was observed (Fig. 2).

### Assessment of vascular features by the examiners

Cochran’s Q test revealed a significant difference in the frequency of occurrence among the five criteria in both healthy tissue and tumor-containing sequences for all examiners (heathy tisse: Q = 50.48; 41.64; 68.48, *p* < 0.001; tumor: Q = 38.15; 34.0; 26.15, *p* < 0.001). Therefore, pairwise comparisons were performed using McNemar’s test. In healthy tissue, vascular characteristics 1 and 2 were consistently observed more frequently than characteristics 3, 4, and 5 by all three reviewers. In tumor tissue, characteristics 4 and 5 were detected significantly more often than 1 and 2 by all reviewers (all *p* < 0.05). Characteristic 3 was identified significantly more frequently than characteristic 1 by all reviewers, but only by two of three reviewers when compared with characteristic 2. The exact frequencies are shown in Table 2.

**Table 2:** Frequency of the criteria. Frequency of occurrence of the respective criteria in healthy tissue and BCC for the individual raters

Criterion	Healthy tissue	BCC
**1**	17/16/19	1/0/0
**2**	19/15/18	2/0/4
**3**	1/0/0	11/5/5
**4**	5/5/1	17/15/14
**5**	2/1/0	14/10/12

These findings suggest that characteristics 1 and 2 are particularly suitable for identifying vessels of healthy tissue, whereas characteristics 4 and 5, and to a lesser extent characteristic 3, serve as reliable indicators of tumor vessels. Detailed p-values for all pairwise comparisons are provided in Tables 3 and 4.

**Table 3:**
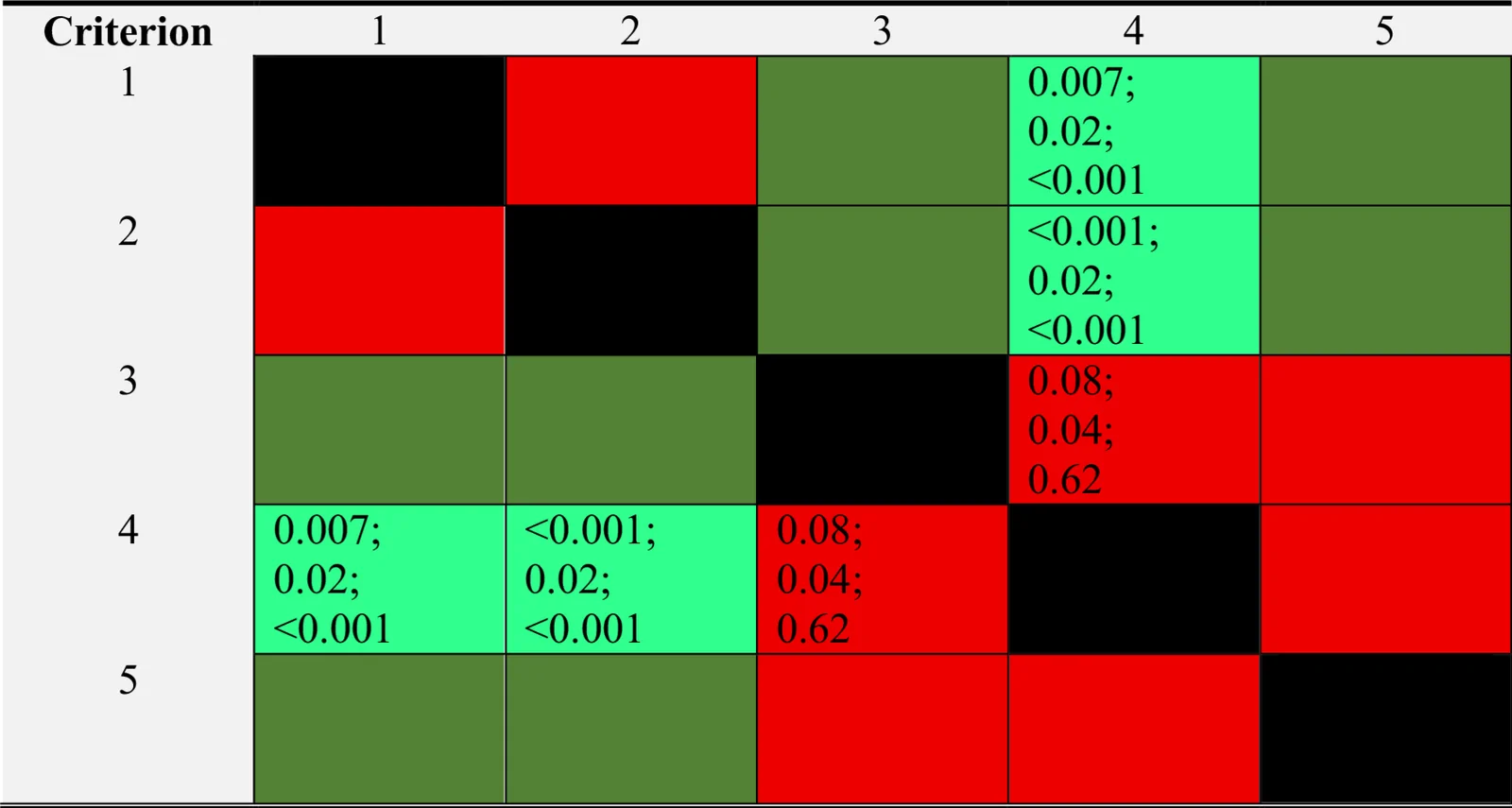
Blinded evaluation of the criteria in healthy tissue. P-values for the three reviewers in the post-hoc group comparison (McNemar’s test). Significance levels were color-coded: red corresponds to *p* ≥ 0.05, while three shades of green (light to dark) represent *p* < 0.05, *p* < 0.01, and *p* < 0.001, respectively. In cases where reviewers reported differing values, the exact p-values were provided. The color coding was assigned according to the least favorable level of significance

**Table 4:**
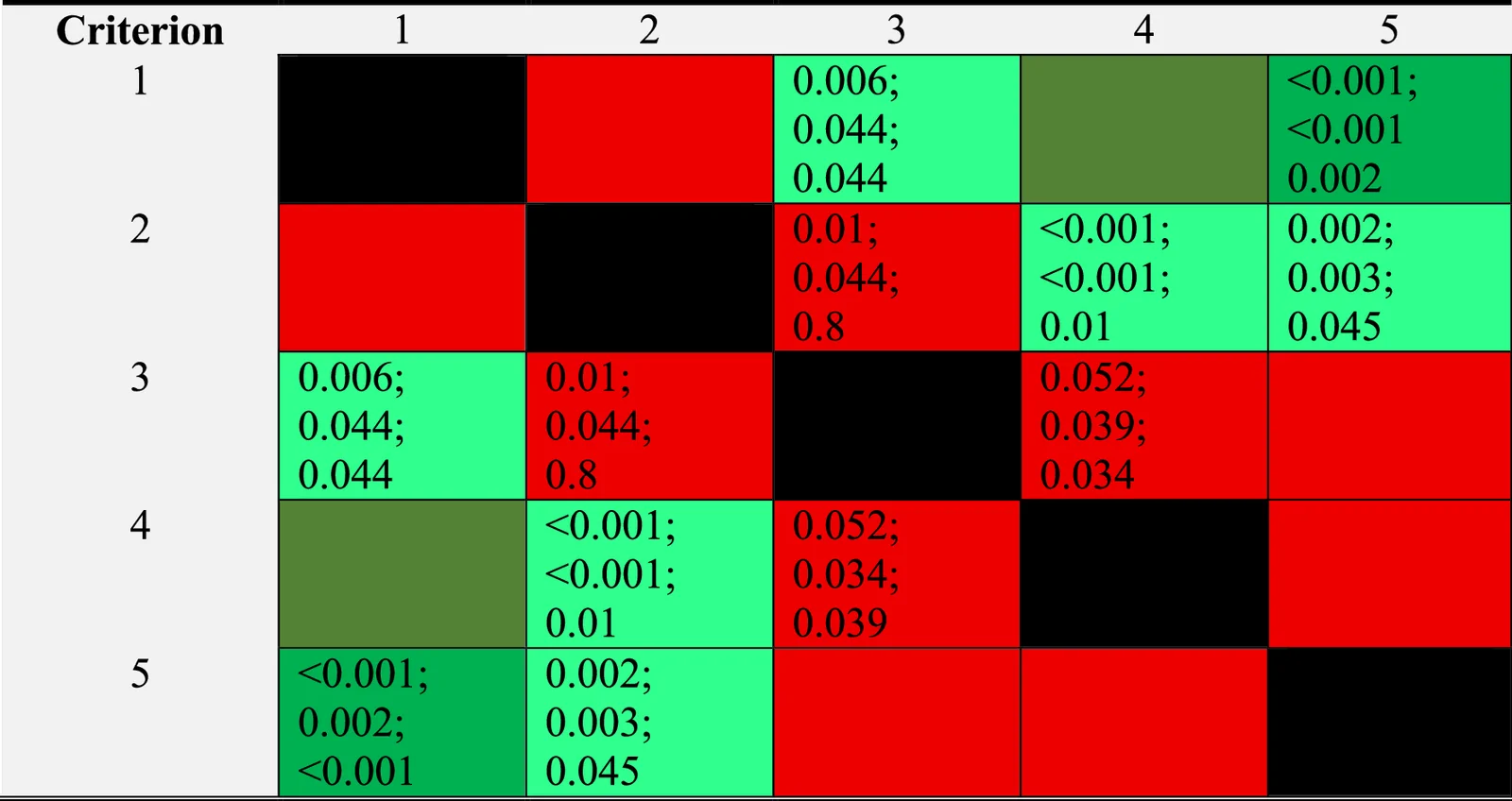
Blinded evaluation of the criteria in BCC. P-values for the three reviewers in the post-hoc group comparison (McNemar’s test). Significance levels were color-coded: red corresponds to *p* ≥ 0.05, while three shades of green (light to dark) represent *p* < 0.05, *p* < 0.01, and *p* < 0.001, respectively. In cases where reviewers reported differing values, the exact p-values were provided. The color coding was assigned according to the least favorable level of significance

### Blinded CLE evaluation

In this study, investigators were able to reliably distinguish vessels from healthy tissue and BCC. Sensitivities of 85%, 95%, and 95% and specificities of 90%, 85%, and 95% were achieved. This resulted in an average sensitivity of 91.7%±4.7 and an average specificity of 86.7%±6.2 (Table 5).

**Table 5:** Results of the blinded evaluation. All reviewers reliably distinguished BCC from healthy tissue

	Accuracy	Sensitivity	Specificity	Ppv	Npv
Examiner 1	82.5	85	80	81	84.2
Examiner 2	90	95	85	86.4	94.4
Examiner 3	95	95	95	95	95
Average	89.2 ± 5.1	91.7 ± 4.7	86.7 ± 6.2	87.5 ± 5.8	91.2 ± 5

With respect to interrater agreement, substantial concordance was observed among the three investigators, with a Fleiss’ kappa of 0.77.

## Discussion

Vascular characteristics have previously been proposed as diagnostic criteria to differentiate BCC from normal skin using a variety of imaging modalities other than CLE [12–15]. In line with these reports, the present study demonstrated that vessels in BCC exhibit a distinct morphology compared to healthy tissue, with greater heterogeneity in distribution and caliber as well as irregular, dilated, and leaky vessel courses. Comparable findings have been described with other techniques [12–15]. For instance, ultrasound, particularly Doppler ultrasound, has been shown to distinguish BCC from normal skin based on disorganized vascular structures and accelerated blood flow [12].

Reflectance confocal microscopy (RCM), a technique related to CLE, identifies branching vascular patterns as indicative of tumor neo-angiogenesis and useful in distinguishing BCC from normal tissue [13, 14]. High-definition optical coherence tomography (OCT) has likewise reported dilated vessels as a characteristic feature of BCC [15]. Furthermore, LC-OCT, which combines the advantages of confocal microscopy and OCT, has also been successfully applied for the detection and characterization of BCC, with blood vessels described as a characteristic feature of nodular BCC [16]. These approaches, however, are primarily intended for diagnostic assessment of tumor entity and dignity rather than intraoperative margin delineation [1].

In this context, our findings should be considered alongside previous CLE studies of BCC that did not specifically address vascular features, as well as CLE investigations of other tumor entities. Compared with initial evaluations of CLE in BCC, the analysis based exclusively on vascular patterns yielded higher sensitivity (91.7% vs. 86.75%) [4]. Particularly relevant appears the comparison with the evaluation of individual morphological criteria such as cell margins, enlarged cells, epidermal shadowing, palisading/polarization, or fibrosis, which achieved accuracy values ranging between 59.4% and 95.3% [4]. The present results, achieving an accuracy of 89.2%, therefore suggest that vascular patterns may represent a particularly reliable discriminator. Nevertheless, direct comparison is limited, as the datasets are based on different acquisition protocols, with the present study specifically filtering vascular sequences for evaluation.

When placed in the context of CLE investigations on other tumor entities, the results of the present study appear equally encouraging. For example, oral cavity carcinomas have been differentiated from healthy mucosa with CLE at a sensitivity of 90.1% and a specificity of 87.4% [7]. Similarly, for laryngeal and pharyngeal carcinomas, values of 95.1% sensitivity and 86.4% specificity have been reported [9]. An early feasibility study investigating CLE in laryngeal lesions reported a sensitivity of 100% but a markedly lower specificity of 40% [17]. A further study focusing specifically on vascular structures in laryngeal and hypopharyngeal carcinomas examined by CLE provides additional reference [8]. The outcomes achieved here for BCC demonstrate a comparably high sensitivity (90.6% in the analysis of laryngeal and hypopharyngeal carcinomas) while clearly outperforming these studies in terms of specificity (71.3% in the evaluation of laryngeal and hypopharyngeal carcinomas) [8].

Several limitations of the present study should be acknowledged. First, this study represents a pilot analysis with a small sample size. Furthermore, all artifact-free video sequences containing identifiable vessels were included in the analysis. However, the exclusion of sequences with substantial artifacts may have introduced a selection bias. Further studies including artifact-affected sequences and systematically quantifying the impact of artifacts on vascular assessment will therefore be important to evaluate the applicability of these findings in clinical practice. Further studies including artifact-affected sequences and systematically quantifying the impact of artifacts on vascular assessment will therefore be important to evaluate the applicability of these findings in clinical practice. Another limitation regarding clinical translation is that only a single morphological feature was investigated, which is not uniformly distributed throughout the tumor or in healthy tissue. In addition, the retrospective study design necessitates validation in larger, prospectively designed cohorts before CLE can be established as a reliable optical biopsy tool for BCC. Nevertheless, the preliminary findings presented here already provide valuable insights for optimizing clinical application. In routine practice, vascular structures will represent only one of several possible diagnostic criteria to differentiate BCC from normal tissue. At the same time, our results suggest that vessels constitute a particularly sensitive marker. Integration of vascular characteristics into a comprehensive scoring system appears promising. Within such a system, vascular features could be assigned greater weight to fully leverage their sensitivity. The goal of such an approach would be to standardize CLE evaluation and thereby reduce investigator dependency.

## Conclusions

This pilot study provides preliminary evidence that vascular structures may serve as a sensitive feature for distinguishing BCC from healthy tissue and may contribute to the further establishment of CLE as an optical biopsy technique. Future development of a multiparametric scoring system incorporating these findings may help to improve diagnostic accuracy, support intraoperative margin assessment, and standardize CLE evaluation. Prospective clinical studies in larger cohorts will be required to validate these results and define their clinical applicability.

## Data Availability

The data supporting the findings of this study are available from the corresponding author upon reasonable request. Due to the sensitive nature of clinical imaging data and in accordance with institutional and data protection regulations, the datasets are not publicly accessible.
